# Supplementary material for: N-Heterocyclic Carbene Adducts of Alkynyl Functionalized 1,3,2-Dithioborolanes
Source: Molecules. 2019 Apr 30;24(9):1690. doi: 10.3390/molecules24091690 (PMC6539715; doi:10.3390/molecules24091690)
Supplement: Supplementary file 1 [file molecules-24-01690-s001.pdf]

Supporting Information

N-Heterocyclic Carbene Adducts of Alkynyl Functionalized 1,3,2-Dithioborolanes

Richard Böser[a], Lars Denker[a], René Frank,*

[a] Institut für Anorganische und Analytische Chemie

Technische Universität Braunschweig

Hagenring 30, 38106 Braunschweig (Germany)

Tel.: (+49) 531-391-5308

E-mail: r.frank@tu-bs.de

Contents

Supporting Information 1

Synthetic Details and Procedures 3

Analytical Methods...3

Synthesis of NHC-Adducts **8** 4

Synthesis of Alkyne Substituted NHC-Adducts **9** 5

NMR Spectra...7

IR spectra 12

X-ray Analysis 13

Synthetic Details and Procedures

General Remarks: All procedures involving air or moisture sensitive compounds were performed under dry argon atmosphere using Schlenk techniques or in a glove box (MBraun 200B). Solvents were purified and dried using a solvent purification system (Braun) and stored over molecular sieve (3-4 Å). All commercially available compounds (TCI, abcr, deutero, Sigma Aldrich) were used without further purification. The compounds $ClBS_2C_2H_4$ (**6**),[i] IiPr (**7a**),[ii] and IMes (**7b**)[iii] were prepared according to literature methods.

Analytical Methods

NMR spectra were recorded on Bruker Avance II-300, Avance III-HD, Avance III-400, and AVII-600. The chemical shifts (δ) are reported in parts per million (ppm). The residual solvent peak ($CHCl_3$, δ = 7.26 ppm) was used for referencing of the ^{1}H spectra. The ^{13}C spectra were internally calibrated by using the ^{13}C resonances of the solvent peaks ($CDCl_3$, δ = 77.16 ppm). For ^{11}B NMR, an external calibration with $BF_3 \cdot Et_2O$ was used. Coupling constants are stated in Hertz (Hz), multiplicities are defined as br (broad), s (singlet), d (doublet), t (triplet), q (quartet), sept (septet), dd (doublet of doublets), dsept (doublet of septets), tt (triplet of triplets), tq (triplet of quartets), ddd (doublet of doublet of doublets), qdq (quartet of doublet of quartets) or m (multiplet). If necessary, 2D NMR experiments (H,C-HSQC, H,C-HMBC) or gated-$^{13}C\{^{1}H\}$ NMR experiments were used to aid in assigning the signals.

Elemental analyses were accomplished by combustion and gas chromatographic analysis using a VarioMICRO Tube and HW detection. Values are reported in %.

GC-MS were recorded on a Finnigan MAT 8400-MSS I, Finnigan MAT 4515 using the EI method and are reported in the form: observed m/z ratio [interpretation].

IR spectra were recorded on a Bruker Vertex 70. IR bands are reported in cm^{-1} and are classified as w (weak), m (medium), and s (strong).

Synthesis of NHC-Adducts 8

Compound 8a

I*i*Pr (**7a**, 400 mg, 2.63 mmol) in toluene (50 mL) was added dropwise to a solution of chloro dithioborolane (**6**, 450 mg, 3.28 mmol) in toluene (25 mL) at 0 °C. After stirring for 10 min at room temperature, the reaction mixture was dried in vacuo. The rose solid was dissolved in THF (5 mL) and precipitated with pentane (20 mL). After filtration and drying *in vacuo* the product was obtained as a light rose solid (65% yield). Crystals suitable for X-ray crystallography were obtained by slow gas phase diffusion of hexane into a solution of the product in toluene.

^{1}H NMR (500.3 MHz, $CDCl_3$): δ (ppm) = 7.09 (s, 2 H, NC*H*), 5.79 (sept, 2 H, C*H*$(CH_3)_2$, $^3J_{HH}$ = 6.7 Hz), 3.12 (s, 4 H, CH_2), 1.48 (d, 12 H, CH_3, $^3J_{HH}$ = 6.7 Hz).

^{13}C{^{1}H} NMR (125.8 MHz, $CDCl_3$): δ (ppm) = 156.3 (m, N*C*N), 117.4 (s, N*C*H), 51.4 (s, *C*$(CH_3)_2$), 37.5 (s, *C*H_2), 23.7 (s, *C*H_3).

^{11}B{^{1}H} NMR (160.5 MHz, $CDCl_3$): δ (ppm) = 4.6 (s, $\omega_{½}$ = 45 Hz).

Elemental Analysis: Calcd. for $C_{11}H_{20}BClN_2S_2$: C 45.45, H 6.94, N 9.64, S 22.06. Found: C 45.58, H 6.97, N 9.42, S 22.15.

Compound 8b

IMes (**7b**, 1.61 g, 5.28 mmol) in toluene (15 mL) was added to a solution of chloro dithioborolane (**6**, 730 mg, 5.28 mmol) in toluene (10 mL) at 0 °C. A bright yellow solid precipitated. The reaction mixture was stirred for 30 min at room temperature. After filtration, washing with toluene (30 mL) and subsequent drying *in vacuo*, the product was obtained as a white solid (93% yield). If required, further purification can be performed by slow diffusion of pentane into a solution of the crude product in $CHCl_3$.

^{1}H NMR (600.1 MHz, $CDCl_3$): δ (ppm) = 7.10 (s, 2 H, NC*H*), 7.01 (s, 4 H, Mes-C*H*), 2.77 (s, 4 H, CH_2), 2.36 (s, 6 H, Mes-*para*-CH_3), 2.21 (s, 12 H, Mes-*ortho*-CH_3).

^{13}C{^{1}H} NMR (150.9 MHz, $CDCl_3$): δ (ppm) = 158.4 (m, N*C*N), 140.7 (s,Mes-*ipso*-C), 136.1 (s, Mes-*para-C*), 133.5 (s, Mes-*ortho-C*), 129.4 (s, Mes-*meta*-*C*H), 123.3 (s, N*C*H), 37.3 (s, *C*H_2), 21.4 (s, Mes-*para*-*C*H_3), 18.5 (s, Mes-*ortho*-*C*H_3).

^{11}B{^{1}H} NMR (128.5 MHz, $CDCl_3$): δ (ppm) = 5.5 (s, $\omega_{½}$ = 80 Hz).

Elemental Analysis: Calcd. for $C_{23}H_{28}BClN_2S_2$: C 62.38, H 6.37, N 6.33, S 14.48. Found: C 62.04, H 6.16, N 5.89, S 14.25.

Synthesis of Alkyne Substituted NHC-Adducts 9

Compound 9a

Ethynyl magnesium bromide (H−C≡C−MgBr, 1.52 mL, 0.5 M in THF, 0.76 mmol) was added dropwise to a solution of adduct **8a** (200 mg, 0.69 mmol) in THF (20 mL) at 0 °C. After stirring for 15 min at 0 °C and 1 h at room temperature the reaction mixture was dried *in vacuo*. The remaining solids were dissolved in DCM (20 mL) and washed with distilled water (3 x 20 mL). The organic phase was dried over $MgSO_4$, filtrated, and dried to yield the product as a light beige solid (75%). Crystals suitable for X-ray crystallography were obtained by storing a solution of the product in toluene at –30 °C.

^{1}H NMR (500.3 MHz, $CDCl_3$): δ (ppm) = 7.05 (s, 2 H, NC*H*), 6.07 (sept, 2 H, C*H*(CH_3)$_2$, $^3J_{HH}$ = 7.0 Hz), 3.22-2.94 (m, 4 H, C*H$_2$*), 2.58 (s, 1H, CC*H*), 1.46 (d, 12 H, C*H$_3$*, $^3J_{HH}$ = 7.0 Hz).

^{13}C{gated-^{1}H} NMR (125.8 MHz, $CDCl_3$): δ (ppm) = 158.1 (br q, N*C*N, $^1J_{BC}$ = 62.2 Hz, $^1J_{BC}$ = 73.8 Hz), 117.3 (ddd, N*C*H, $^1J_{CH}$ = 194.7 Hz, $^2J_{CH}$ = 11.2 Hz, $^3J_{CH}$ = 4.7 Hz), 99.2 (m, *C*CH), 85.9 (br d, C*C*H, $^1J_{CH}$ = 238.7 Hz), 50.4 (dsept, *C*(CH_3)$_2$, $^1J_{CH}$ = 144.6 Hz, $^2J_{CH}$ = 4.2 Hz), 38.0 (tt, *C*H_2, $^1J_{CH}$ = 140.3 Hz, $^2J_{CH}$ = 2.1 Hz), 23.7 (qdq, *C*H_3, $^1J_{CH}$ = 127.9 Hz, $^2J_{CH}$ = 4.5 Hz, $^3J_{CH}$ = 4.5 Hz).

^{11}B{^{1}H} NMR (160.5 MHz, $CDCl_3$): δ (ppm) = −11.1 (s, $\omega_{½}$ = 19 Hz).

Elemental Analysis: Calcd. for $C_{13}H_{21}BN_2S_2$: C 55.71, H 7.55, N 10.00, S 22.88. Found: C 56.03, H 7.68, N 10.14, S 22.74.

MS (EI): m/z = 280.12 [M]$^+$, 128.0 [M−I*i*Pr]$^+$.

IR (ATR, cm^{-1}): diagnostic band $\tilde{\nu}$ = 3306 [w, ν(CC-H)].

Compound 9b

Ethynyl magnesium bromide (H−C≡C−MgBr, 6.4 mL, 0.5 M in THF, 3.2 mmol) was added dropwise to a solution of adduct **8b** (1.28 g, 2.9 mmol) in THF (80 mL) at 0 °C. After stirring for 30 min at 0 °C and 1 h at room temperature the reaction mixture was dried *in vacuo*. The remaining solids were dissolved in DCM (150 mL) and washed with distilled water (3 x 150 mL). The organic phase was dried over $MgSO_4$, filtrated, and dried to yield the product as a beige solid (71%). Crystals suitable for X-ray crystallography were obtained by gas phase diffusion of pentane into a solution of the product in $CDCl_3$.

^{1}H NMR (600.1 MHz, $CDCl_3$): δ (ppm) = 7.00 (s, 2 H, NC*H*), 6.96 (s, 4 H, Mes-C*H*), 2.77-2.37 (m, 4 H, C*H$_2$*), 2.33 (s, 6 H, Mes-*para*-C*H$_3$*), 2.21 (s, 12 H, Mes-*ortho*-C*H$_3$*), 2.04 (s, 1 H, CC*H*).

$^{13}C\{gated-^{1}H\}$ NMR (150.9 MHz, $CDCl_3$): δ (ppm) = 161.8 (br q, N*C*N, $^{1}J_{BC}$ = 61.0 Hz, $^{1}J_{BC}$ = 72.8 Hz), 141.1 (q, Mes-*ipso*-C, $^{3}J_{CH}$ = 6.0 Hz), 139.7 (q, Mes-*para-C,* $^{2}J_{CH}$ = 6.0 Hz), 135.5 (q, Mes-*ortho-C,* $^{2}J_{CH}$ = 6.0 Hz), 128.7 (m, Mes-*meta*-*C*H), 124.4 (dd, N*C*H, $^{1}J_{CH}$ = 198.5 Hz, $^{2}J_{CH}$ = 11.4 Hz), 97.4 (m, *C*CH), 85.6 (d, C*C*H, $^{1}J_{CH}$ = 234.8 Hz), 37.3 (tt, *C*H_2, $^{1}J_{CH}$ = 140.1 Hz, $^{2}J_{CH}$ = 2.8 Hz), 20.9 (tq, Mes-*para*-*C*H_3, $^{1}J_{CH}$ = 126.5 Hz, $^{3}J_{CH}$ = 4.7 Hz), 18.2 (m, Mes-*ortho*-*C*H_3).

$^{11}B\{^{1}H\}$ NMR (160.5 MHz, $CDCl_3$): *δ* (ppm) = −11.5 (s, $\omega_{½}$ = 30 Hz).

Elemental Analysis: Calcd. for $C_{25}H_{29}BN_2S_2$: C 69.43, H 6.76, N 6.48, S 14.83. Found: C 68.98, H 6.77, N 6.39, S 14.65.

MS (EI): m/z = 432.19 $[M]^+$, 128.0 $[M−IMes]^+$.

IR (ATR, cm^{-1}): diagnostic bands $\tilde{\nu}$ = 3225 [w, ν(CC-H)], 2044 [w, ν(C≡C)].

NMR Spectra

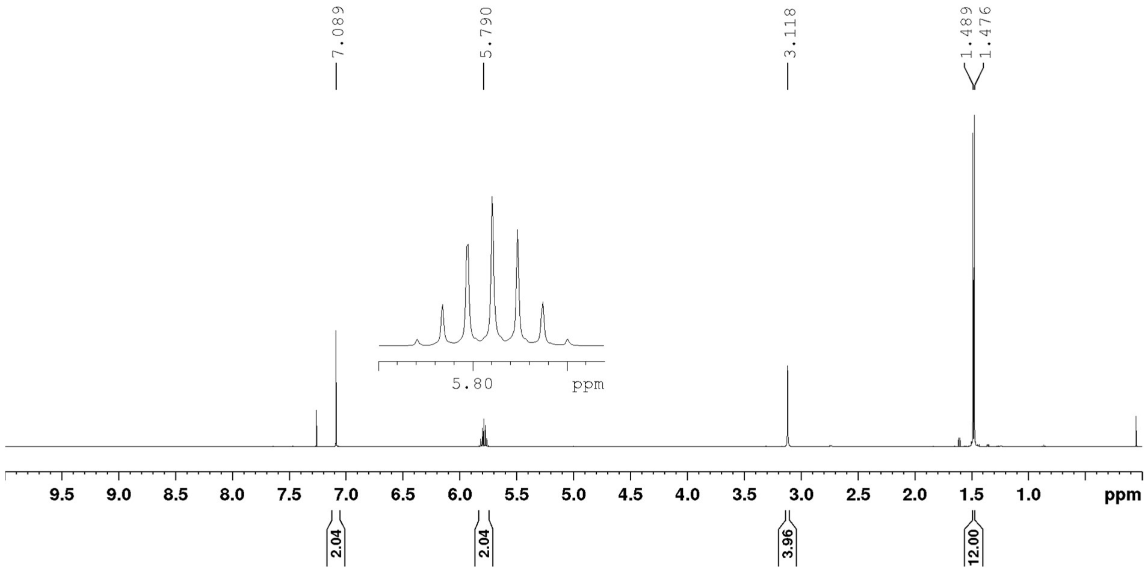

Figure 1. ^{1}H NMR spectrum of **8a** (500.3 MHz, $CDCl_3$, 25 °C).

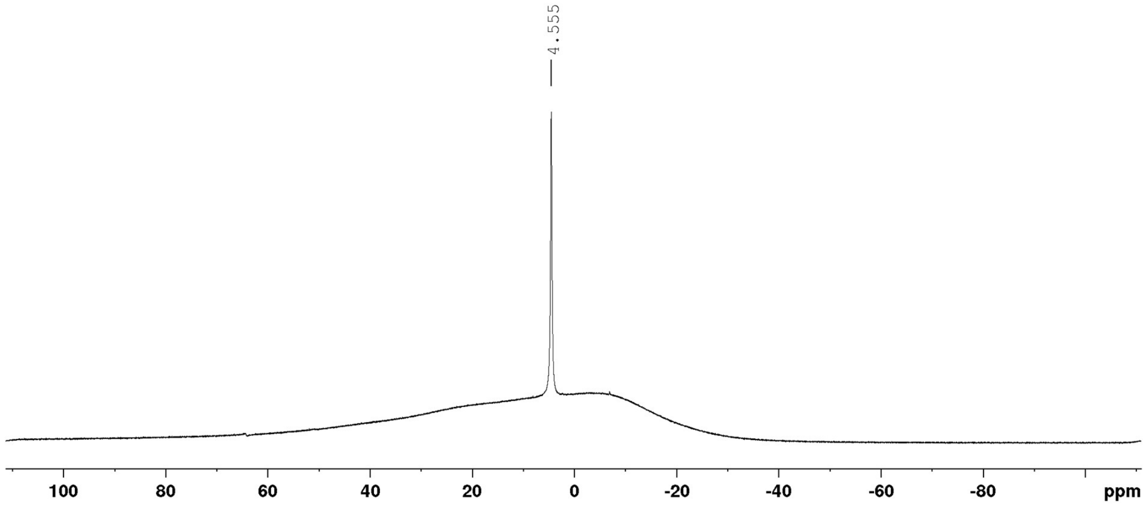

Figure 1: 11B{1H} NMR spectrum of 8a (160.5 MHz, CDCl3, 25 °C).

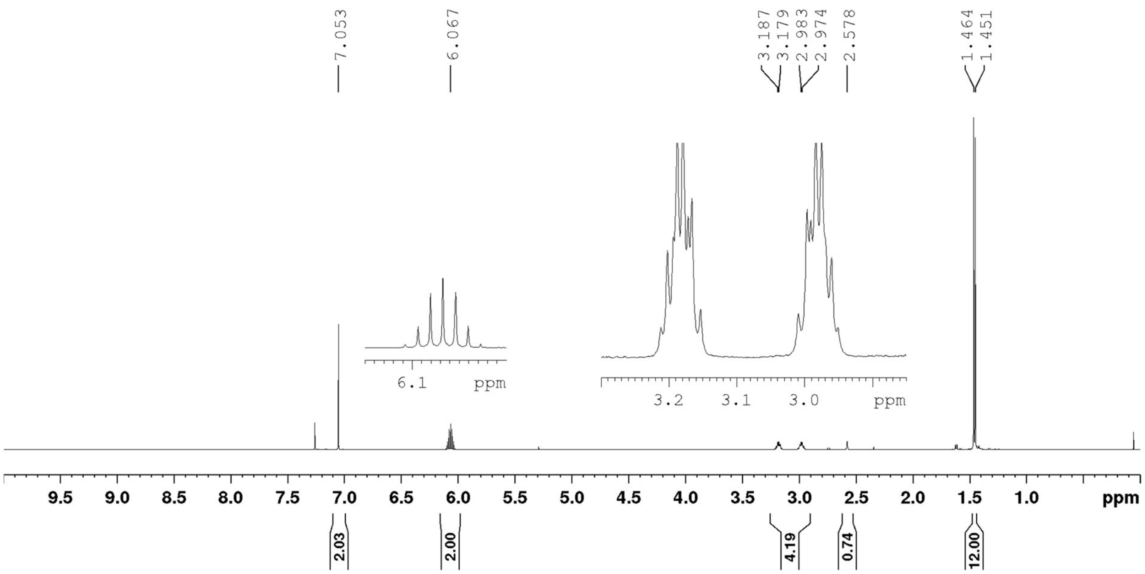

Figure 2: 1H NMR spectrum of 9a (500.3 MHz, CDCl3, 25 °C).

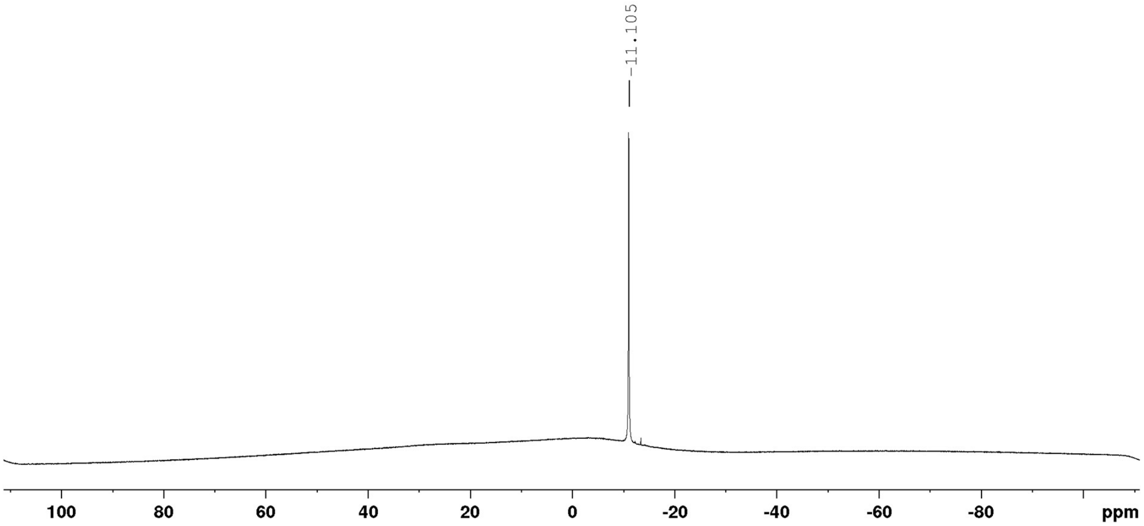

Figure 3: 11B{1H} NMR spectrum of 9a (160.5 MHz, CDCl3, 25 °C).

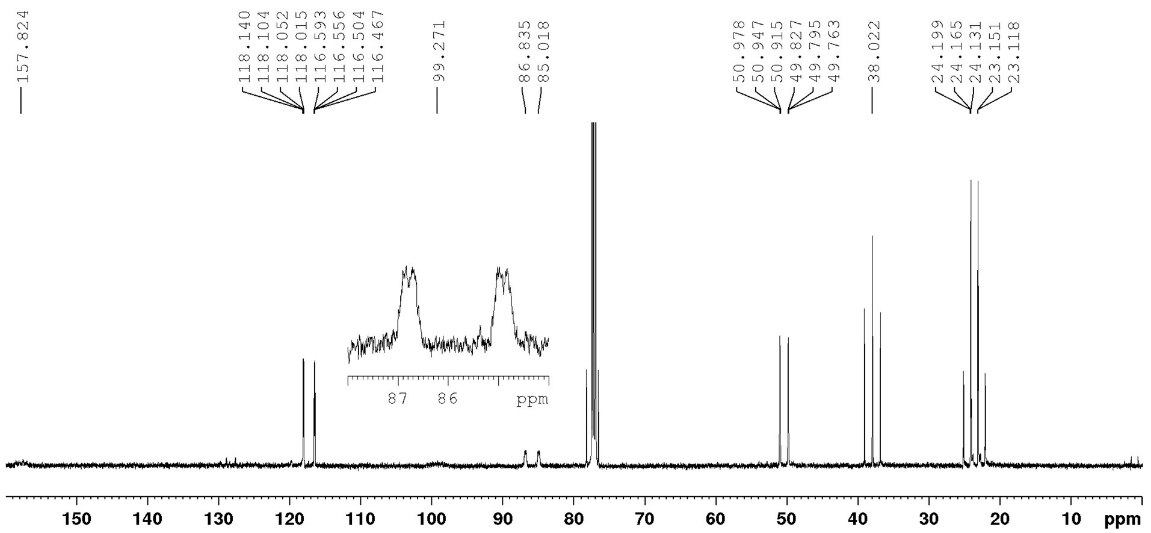

Figure 4: Gated-13C{1H} NMR spectrum of 9a (125.8 MHz, CDCl3, 25 °C).

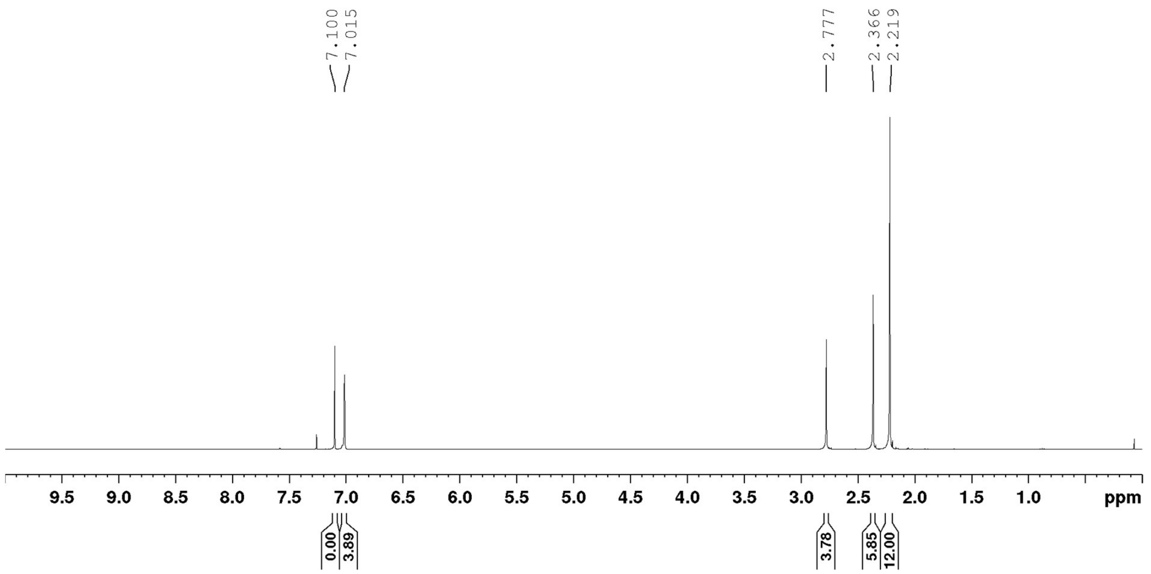

Figure 5: 1H NMR spectrum of 8b (400.4 MHz, CDCl3, 25 °C).

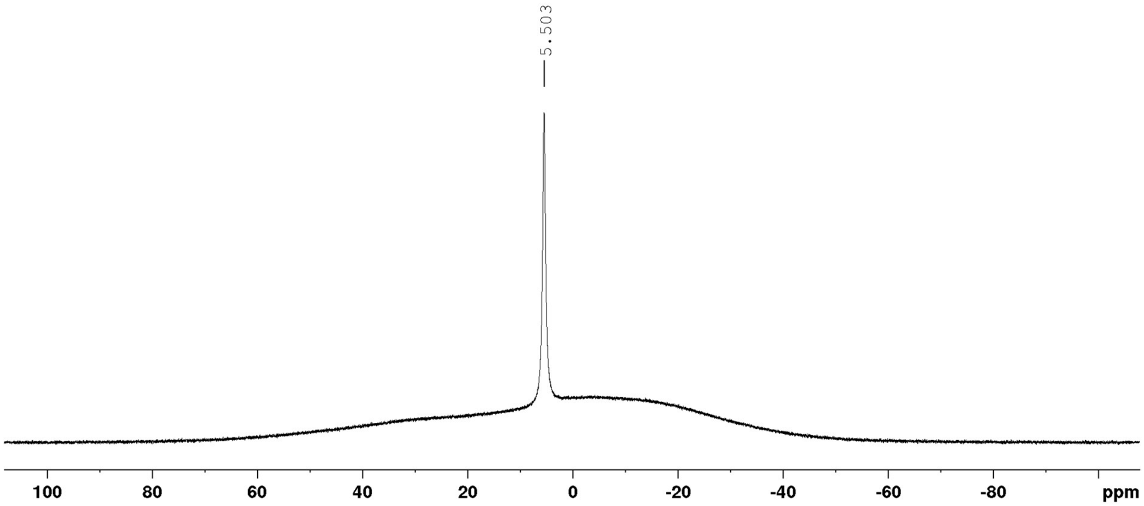

Figure 6: 11B{1H} NMR spectrum of 8b (128.5 MHz, CDCl3, 25 °C).

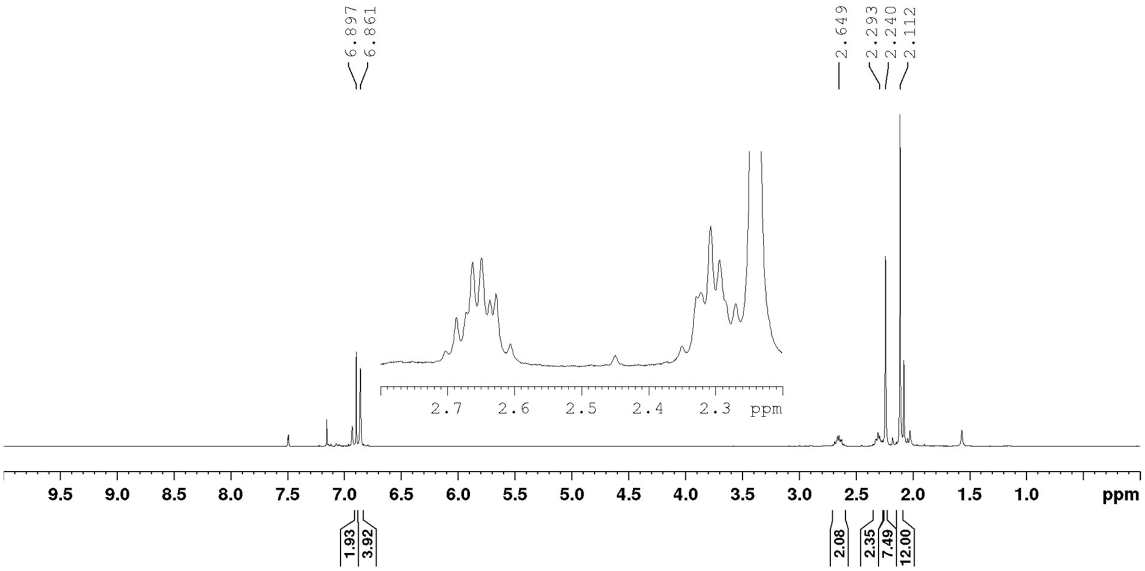

Figure 7: 1H NMR spectrum of 9b (300.3 MHz, CDCl3, 25 °C).

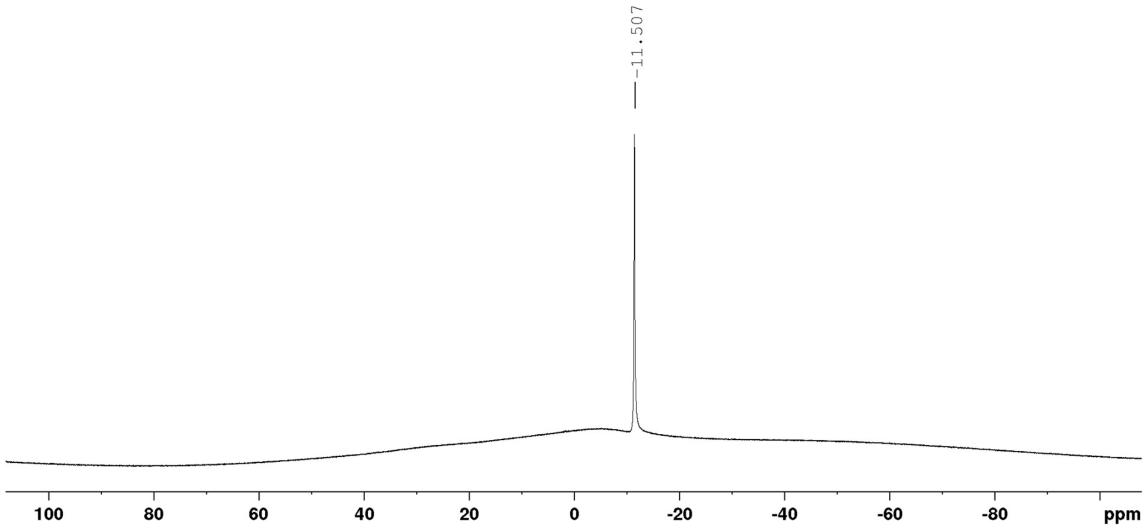

Figure 8: 11B{1H} NMR spectrum of 9b (160.5 MHz, CDCl3, 25 °C).

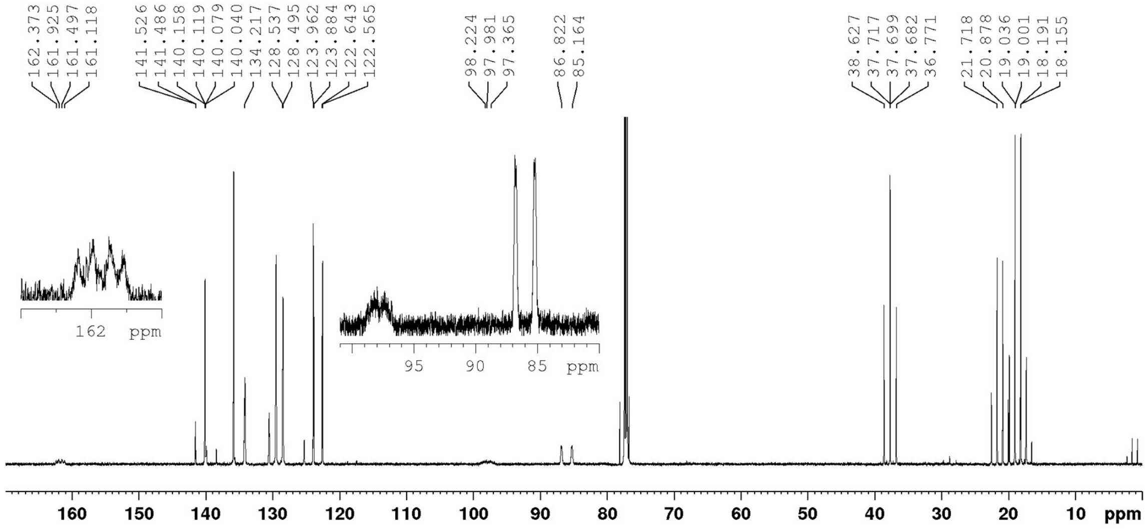

Figure 9: Gated-13C{1H} NMR spectrum of 9b (150.9 MHz, CDCl3, 25 °C).

IR spectra

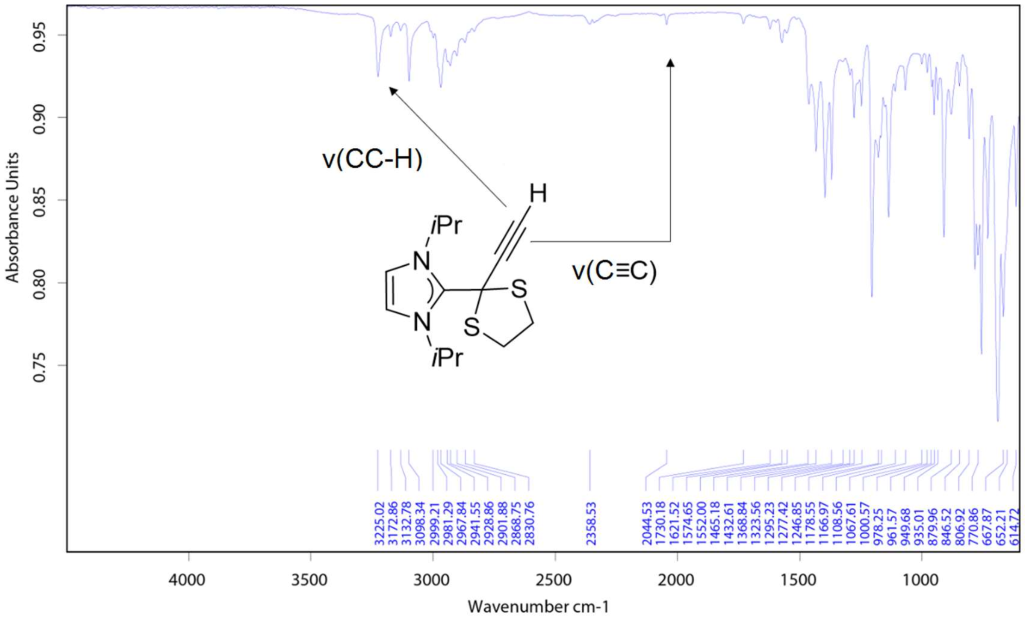

Figure 10: IR spectrum of 9a showing the stretching frequencies of the alkyne moiety.

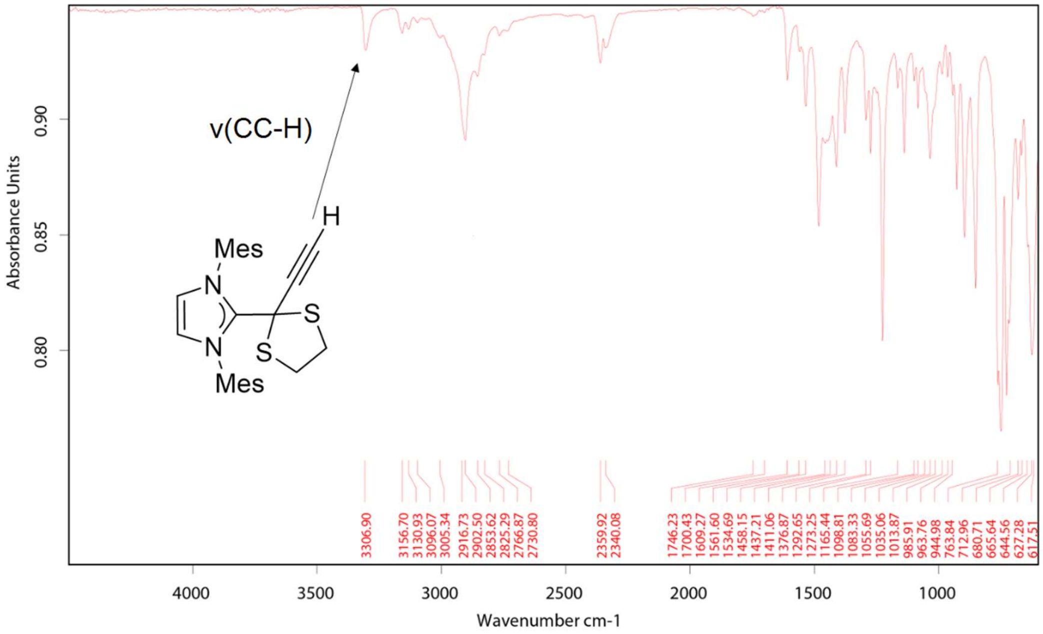

Figure 11: IR spectrum of 9b showing the stretching frequencies of the alkyne moiety.

X-ray Analysis

Data collections were performed by mounting single crystals on glass fibers or MiTeGen mounts in perfluorinated oil. Diffractometers used for intensity measurements (at 100 K) were Oxford Diffraction Nova A with Mo *K*α radiation or Rigaku XtaLAB Synergy S Single Source with Cu *K*α. Absorption correction was applied based on multi-scan methods. Data reduction was performed using the program CrysalisPro.[iv] The structures were solved with SHELXT-14/5[v] and refined anisotropically on F^2 using the programs SHELXL-14/7 and SHELXL-17/1.[vi]

Treatment of hydrogen atoms: The terminal alkyne hydrogen atom in the structures of **9a** and **9b** were refined freely. The remaining hydrogen atoms were placed in idealized positions and refined using a riding model.

Compounds **8a** and **9a**: The co-crystallizing toluene solvent molecules were located at the center of inversion and were disordered over two positions with a refined occupancy of 1:1. They were placed using disordered structure refinement (DSR)[vii] utilizing the Olex[viii] FragmentDB tool and refined with a discrete disorder model.

Compound **8a**: The simulated precession images (see Figure 11) showed unindexed peaks with low intensity suggesting a modulation in the *b*-axis, but quadruplication of the *b*-axis did not lead to a meaningful integration of the reflexes. Therefore, these small peaks were ignored and the crystal structure was solved as a non-modulated structure.

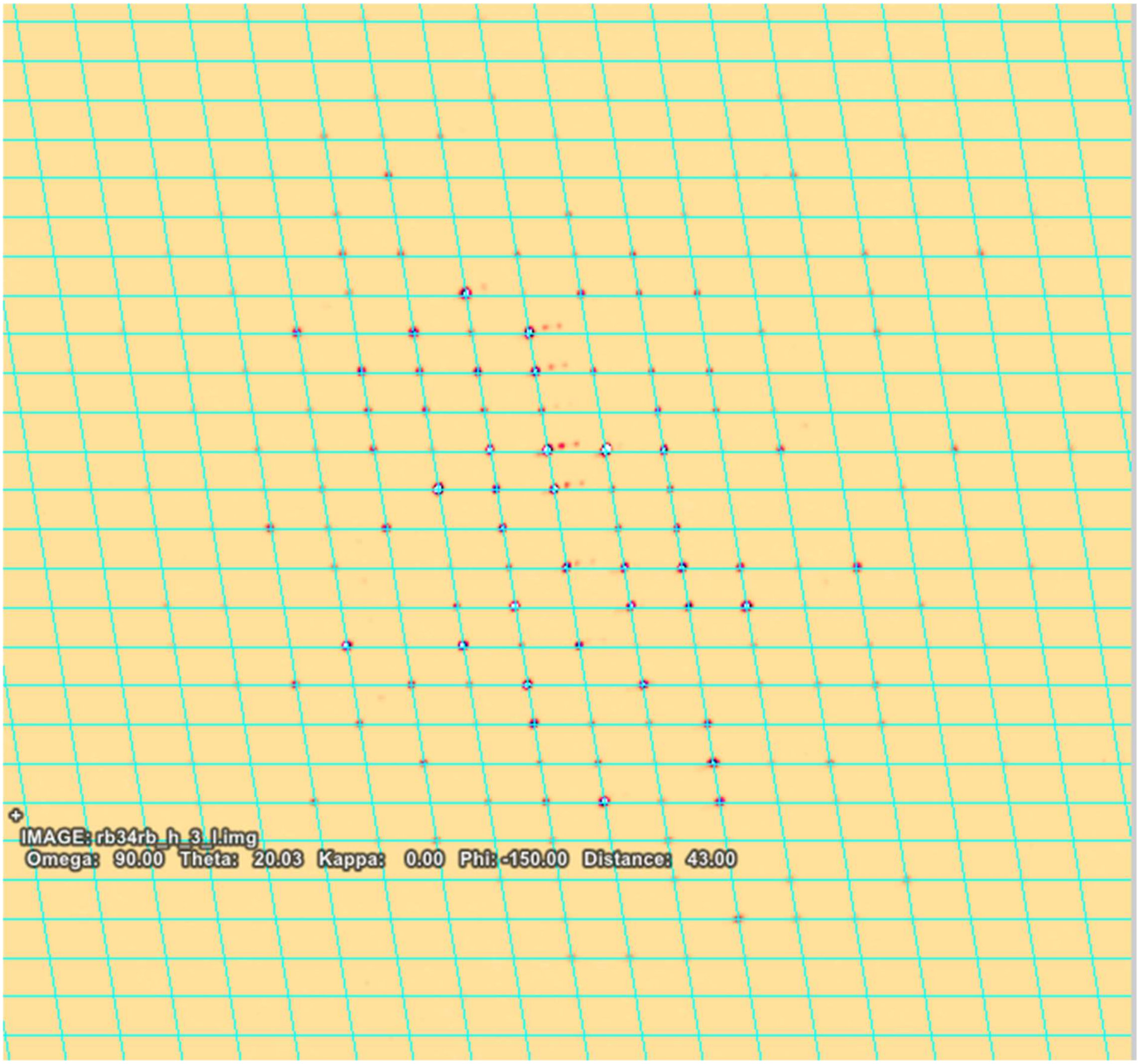

Figure 12: Simulated precession image of the h 3 l layer showing the weak unindexed peaks of the data for compound 8a.

Complete data have been deposited at the Cambridge Crystallographic Data Centre (CCDC) under the CCDC numbers 1907368 (**8a**), 1907369 (**9a**), and 1907370 (**9b**), and can be obtained free of charge at http://www.ccdc.cam.ac.uk/.

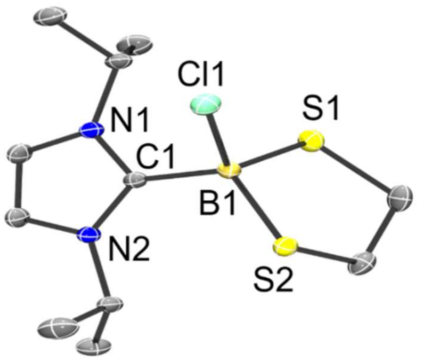

Figure 13: Molecular structure of compound 8a. Ellipsoids drawn at the 50% probability level. Hydrogen atoms and toluene are omitted for clarity. Selected bond lengths (Å) and angles (°): B1–C1 1.6298(18), B1–S1 1.9198(14), B1–S2 1.9018(14), B1–Cl1 1.9074(14), C1–B1–Cl1 105.98(9), C1–B1–S1 106.65(8), C1–B1–S2 119.60(9), N2–C–-B1–S1 113.01(13).

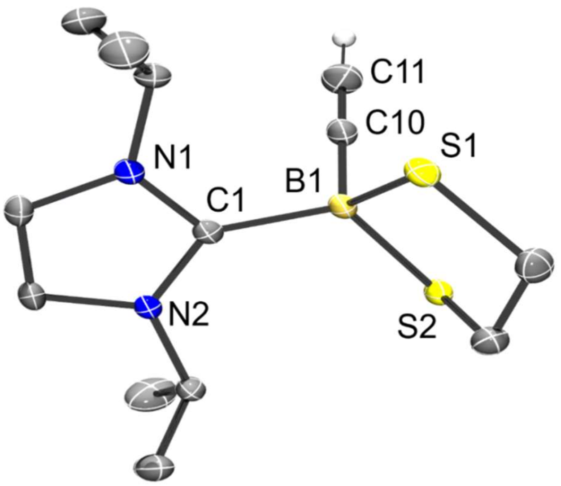

Figure 14: Molecular structure of compound 9a. Ellipsoids drawn at the 50% probability level. Hydrogen atoms of minor relevance and toluene are omitted for clarity. Selected bond lengths (Å) and angles (°): C1-B1 1.6427(15), B1-S1 1.9140(12), B1-S2 1.9642(12), B1-C10 1.5754(16), C10-C11 1.1975(18), C1-B1-C10 109.04(9), C1-B1-S1 118.69(7), C1-B1-S2 106.20(7), N1-C1-B1-C10 124.80(12).

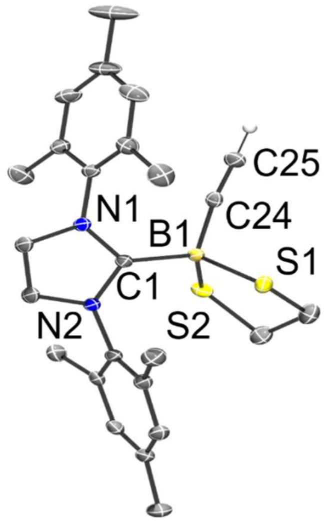

Figure 15: Molecular structure of compound 9b. Ellipsoids drawn at the 50% probability level. Hydrogen atoms of minor relevance and CDCl3 are omitted for clarity. Selected bond lengths (Å) and angles (°): B1-C1 1.6335(19), B1-S1 1.9397(15), B1-S2 1.9343(15), B1-C24 1.5811(19), C24-C25 1.201(2), C1-B1-C24 113.14(11), C1-B1-S1 113.13(9), C1-B1-S2 108.08(9), N1-C1-B1-C24 176.64(12).

Table 1: Selected crystallographic data for compounds 8a, 9aʃ and 9b; measured by X-ray structure analysis.

	Compound **8a**	Compound **9a**	Compound **9b**
CCDC	1907368	1907369	1907370
Formula	$C_{14.50} H_{24} B Cl N_2 S_2$	$C_{16.57} H_{25.08} B N_2 S_2$	$C_{26} H_{29} B Cl_3 D N_2 S_2$
M [g mol^{-1}]	336.74	327.30	552.80
T/K	100(2)	100(2)	100(2)
λ/Å	0.71073	1.54184	0.71073
Crystal system	Triclinic	Triclinic	Monoclinic
Space group	P -1	P -1	*P*2$_1$/*c*
a/Å	7.9436(6)	7.0536(2)	11.25800(10)
b/Å	9.7731(6)	7.8730(2)	16.89950(10)
c/Å	12.3213(8)	18.4532(2)	15.4788(2)
α/°	70.113(6)	85.689(2)	90
β/°	79.241(6)	84.420(2)	107.7710(10)
γ/°	83.504(4)	63.911(2)	90
V/Å^3	882.42(11)	915.39(4)	2804.39(5)
Z	2	2	4
D_x/g cm^{-3}	1.267	1.187	1.309
μ/mm^{-1}	0.446	2.586	0.494
F(000)	358	351	1152
Cryst. size/mm	0.25x0.15x0.12	0.22x0.12x0.05	0.54x0.30x0.22
Theta range/°	2.219 to 31.009	2.407 to 77.546	2.250 to 29.129
Reflections collected	57269	38327	143391
Indep. reflections	5302	3823	7548
R(int)	0.0476	0.0366	0.0406
Data / restraints / parameters	5302 / 122 / 222	3823 / 122 / 236	7548 / 0 / 317
GooF	1.048	1.069	1.033
R1 (F, >4σ(F))	0.0344	0.0295	0.0365
wR2 (F2, all refl.)	0.0816	0.0799	0.0993
max. Δρ/e Å^{-3}	0.520/−0.237	0.310/−0.292	0.870/−0.435

[i] A. Finch, J. Pearn, *Tetrahedron*, **1964**, *20*, 173−176.
[ii] T. Schaub, M. Backes, U. Radius, *Organometallics*, **2006**, *25*, 4196−4206.
[iii] A. J. Arduengo, R. Krafczyk, R. Schmutzler, *Tetrahedron*, **1999**, *55*, 14523−14534.
[iv] Rigaku Oxford Diffraction, *CrysAlisPRO Software System* version 1.171.39.46 (**2018**), Rigaku Corporation, Oxford, UK.
[v] G. M. Sheldrick, *Acta Cryst.,* **2015**, *A71*, 3−8.
[vi] G. M. Sheldrick, *Acta Crystallogr., Sect. A: Found. Crystallogr.* **2008**, *64*, 112–122.
[vii] D. Kratzert, J. J. Holstein, I. Krossing, *J. Appl. Cryst*., **2015**, *48*, 933−938.
[viii] O.V. Dolomanov, L.J. Bourhis, R.J. Gildea, J.A.K. Howard, H. Puschmann, *J. Appl. Cryst.,* **2009**, *42*, 339−341.
